# Financing constraints and impact on corporate performance growth: Study in China listed logistics enterprises

**DOI:** 10.1371/journal.pone.0285671

**Published:** 2023-06-28

**Authors:** Ke Liu, Zhaoping Wang, Ran Du, Heng Chen, Yajing Li

**Affiliations:** 1 School of International Business, Shaanxi Normal University, Xi’an, Shaanxi, China; 2 School of Economics, Huazhong University of Science and Technology, Wu Han, Hu Bei, China; 3 School of Management, Xi’an Polytechnic University, Xi’an, Shaanxi, China; University of Almeria, SPAIN

## Abstract

Based on 2010–2019 Chinese logistics listed companies as research samples, the paper used the binary Logit model measuring degree of financing constraints. The Kernel density function and Markov chain model are used to forecast China listed companies financing logistics dynamic constraints and business performance growth. Furthermore the stock of knowledge was chosen as a threshold variable to explore the impact of financing constraints on corporate performance growth of listed logistics enterprises. We find that the degree of financing constraints of logistics enterprises in our country has not been significantly eased. Corporate performance has not changed significantly and there are no obvious spatial gap and polarization with the passage of time. The impact of financing constraints on the corporate performance growth of logistics enterprises in China has a double threshold effect of knowledge stock, and has an inhibitory effect that first increases and then decreases. This is because in the short term, the investment of knowledge stock by enterprises can crowd out more corporate liquidity, and in the long run, it is related to the conversion rate of the knowledge stock itself. Because of the uneven regional distribution of resources and differences in the degree of economic development, there is a growing disincentive effect in central China as the stock of knowledge accumulates.

## Introduction

With the vigorous development of global economic integration, the logistics industry has become more important to promote the effective allocation of global resources. At the same time, under the wave of global economic integration, the connectivity of global networks and infrastructure has also been changed. In particular, the improvement of the environment and conditions for the development of the logistics industry has greatly expanded the network space for the development of the logistics industry. The agglomeration of different national factors to more efficient countries or regions has injected new momentum into the economic development of different countries. Although China is a large logistics country, the traditional extensive growth pattern relying on the expansion of factor scale leads to the "Big but not strong" character of China logistics industry. And this low-quality development pattern is non-sustainable.

As the largest developing country, China logistics industry is affected by historical reasons in the process of development. The proportion of private enterprises in the industry is relatively high, resulting in logistics enterprises often being discriminated against by the market when financing in the financial market, which will cause financing difficulties. The proportion of private enterprises in the logistics industry is relatively high, and although the state requires the enhancement of the capacity of financial service entities, the entire logistics industry is likely to be unable to fully cover in the short term. Therefore, financing difficulties remain a major problem for the sustainable development of the logistics industry. And this problem will further lead to the inability of advanced logistics equipment to be effectively introduced and applied, resulting in limited performance growth of logistics enterprises. Therefore, it cannot effectively support the logistics industry to move towards high-quality development.

The existing research on the relationship between corporate financing constraints and enterprise performance growth has been explored by scholars as early as 1950. Modigliani and Miller (1958) argue that in a perfectly competitive capital market, firms can obtain funds from outside in a timely manner according to their needs. Investment behavior is not affected by the company’s financial situation, but only related to the investment needs of the enterprise, in which case there is no financing constraint [1]. However, there are many problems in the real capital market, and almost all of them will have financing constraints in the process of enterprise development. The specific problem of financing constraints was proposed by Fazzari et al. (1988). The author defines financing constraints as that in the capital market, the internal and external financing costs of enterprises in the process of production and operation are quite different, and enterprises cannot bear excessive external financing costs, resulting in insufficient financing [2].

Regarding the research on financing constraints, existing studies focus more on the influencing factors of financing constraints. For example, Li and Liu (2015) analyzed the impact of per capita salary and labor intensity on the degree of financing constraints of enterprises from the perspective of internal factors [3]. Du et al. (2017) analyzed the impact of effort factors on exogenous financing [4]. Ma Zhen (2019) empirically tested the impact mechanism of tax reduction on corporate financing constraints [5]. Tian et al. (2019) examined the impact of environmental performance on financing constraints [6]. Zhao et al. (2019) examined the impact of interest rate liberalization on the financing of R&D investment in microenterprises [7]. Huang and Li (2020) studied the impact of high-tech enterprise identification on external financing constraints of SMEs [8]. Some scholars have analyzed the influencing factors of financing constraints of private enterprises [9, 10]. Yao et al. (2017) studied the impact of operational capability on the financing efficiency of innovative firms [11]. Some scholars believe that the impact of financing constraints on enterprise performance is related to the nature of the industry. For example, Allen (1993) suggested that indirect financing methods such as bank loans are more friendly to traditional lower-risk industries, while innovative enterprises mostly obtain funds through equity financing [12]. Brown et al. (2009) believed that a favorable stock market environment can reduce the financing cost of enterprises, thereby improving the profitability of enterprises [13]. Tian et al. (2017) pointed out that the secondary industry was most affected by the financial crisis, and the primary industry could resist the downward pressure on economic operation [14].

In addition to the above studies, some scholars have pointed out that there is a threshold effect on the impact of financing on enterprises. Li (2021) pointed out that there is a threshold effect of different funds for housing prices and economic growth in the real estate industry [15]. Wang (2018) believed that financing constraints have a double threshold effect on the total factor productivity of Chinese manufacturing enterprises [16]. Lu and Zhao (2019) took enterprise scale and dividend payout ratio as the threshold variables to analyze the panel threshold effect of bridge financing on the performance of Second-board Market enterprises [17]. Most scholars have proposed that the impact of knowledge stock on performance has a catalytic effect. For example, DeCarolis et al. (1999) studied the biotechnology industry and found that when a firm has a larger quantity and higher quality of knowledge, it improves the performance of its new products [18]. Based on the cross-sectional data of 364 technology-based SMEs in Foshan in 2014, Xue (2016) found that market knowledge in the knowledge stock of enterprises has a positive role in promoting the disruptive innovation of SMEs [19]. However, some scholars have empirically found that knowledge stock has a negative impact on innovation performance. When analyzing the Irish questionnaire data, Roper (2015) found that the existing knowledge reserve does not have a positive impact on the innovation output of enterprises, but has a weak negative impact, and reflects negative path dependence or potential core capability rigidity, rather than the accumulation of competitive advantage [20]. Zhou and Chen (2014) took high-tech enterprises in China Pearl River Delta region as a sample, and found that enterprise process inertia and information inertia will affect the knowledge integration of enterprises, thereby negatively affecting the company’s new product development performance [21].

By reorganizing and systematically summarizing relevant research results, we find that the research on financing constraints focuses on small and medium-sized enterprises and private enterprises. In the industry, it mainly focuses on the research of real estate and Internet enterprises, and there is less research on the logistics industry. We also found that the research on logistics companies is mainly focused on the industry level, mainly from the perspective of logistics efficiency, business performance, strategic issues and other perspectives. Most of the existing research conclusions support that financing constraints negatively affect the daily investment and business activities of enterprises. Most studies believe that knowledge stock promotes corporate performance. However, a few scholars have found that the knowledge stock will make enterprises path dependent, which will not be conducive to the improvement of enterprise innovation performance.

At this stage, how strong is the financing constraint facing China logistics industry? And what is the impact of financing constraints on the performance growth of logistics enterprises and how they are affected? Existing studies have paid less attention to this issue. Therefore, the paper selects the data of China logistics listed enterprises and constructs a financing constraint index to measure the degree of financing constraints of China logistics enterprises. The kernel density and Markov chain model are used to analyze and predict the dynamic evolution trend of financing constraints and corporate performance of logistics enterprises in China. The knowledge stock is selected as the threshold variable to analyze the panel threshold effect of financing constraints affecting the performance growth of logistics enterprises. Through the research in the paper, it is hoped that the performance of logistics enterprises can be improved and the adverse impact of financing constraints can be reduced.

Research literature has shown that financing constraints negatively affect corporate performance. If the degree of financing constraint of enterprises is high, the growth of enterprise performance will be slow. The lower the degree of credit constraint of export enterprises, the more external funds they can obtain, thereby improving their financial performance [22]. Increasing equity concentration is conducive to easing the financing constraints of real estate enterprises, thereby alleviating the constraints of financing constraints on enterprise performance [23]. Financing constraints can reduce the quality of Chinese agricultural product export enterprises, which is not conducive to the improvement of corporate profits [24]. Modest and major financing constraints can undermine a company’s environmental performance with high investment costs and biased incentives [6]. Easing financing constraints can improve corporate performance, mainly in the following aspects. First, low financing constraints indicate that enterprises can obtain stable capital guarantees to meet their capital needs for R&D and expansion of reproduction. In particular, Chinese small and medium-sized enterprises have difficulty obtaining bank credit loans, which has led to restrictions on their purchase of equipment and the introduction of talents. Second, when enterprises are under low financing constraints, they can reduce corporate risks and encourage managers to seize opportunities, thereby improving corporate performance. When enterprises are under high financing constraints, managers will hesitate to promote high-risk and critical projects, which may miss opportunities and be detrimental to the operation and development of enterprises. Third, when the enterprise has high financing costs, the public and investors will question the company’s operating ability and development prospects, resulting in a bad reputation for the enterprise. A good governance structure and governance ability, and a relatively good business reputation are conducive to the improvement of corporate profits. Therefore, we propose the following hypothesis.

**H1**: Financing constraints have a restraining effect on the performance growth of China logistics enterprises.

Knowledge stock refers to the total amount of knowledge of an organizational system at a specific point in time, which is the sum of all knowledge attached to the people, equipment and organizational structure within the organizational system [25]. Knowledge stock is a static measurement of the existing knowledge of the enterprise, including enterprise experience, human resources, etc. It can promote the rational allocation and efficient use of resources such as funds and personnel invested in research and development by enterprises. In order to improve the conversion rate of research and development investment [26]. Zhang and Tang (2022) analyzed the threshold effect of knowledge stock between innovation expenditure and performance [27].The knowledge absorptive capacity of the innovation subject, the degree of knowledge spillover, and the price of external knowledge are the decisive factors affecting the acquisition mode of external knowledge [28–30]. This is essentially a difference in knowledge stock. Since the diffusion of knowledge is limited by geographical scope, the localization of knowledge becomes one of the decisive factors affecting the direction of independent research and development investment of innovation subjects. The accumulation of corporate knowledge stock is directly proportional to the efficiency of transforming R&D investment into performance. The level of knowledge stock means the degree of availability of knowledge, and at the same time affects the price of knowledge and the difficulty of acquiring knowledge, thus becoming a decisive factor. Enterprises with different knowledge stocks may have large differences in the absorption and conversion efficiency after acquiring knowledge. Compared with enterprises with less knowledge stock, network infrastructure construction may have a stronger innovation performance improvement effect on enterprises with more knowledge stock [31]. With the increase of enterprise knowledge stock, more funds need to be invested in knowledge in the early stage, so it occupies more enterprise funds, resulting in enterprises facing stronger financing constraints, and then gradually enhancing the inhibition effect on corporate performance growth. However, if an enterprise only invests funds in a small project, it will not occupy more funds, and the maximum capital occupation will occur only after reaching a certain amount. From the perspective of the long-term development of enterprises, the transformation of knowledge stock will alleviate the financing constraints of enterprises to a certain extent. The inhibitory effect of financing constraints on the growth of enterprise performance is gradually decreasing, and even improves enterprise performance. Therefore, we propose the following hypothesis.

**H2**: The impact of financing constraints on the performance growth of logistics enterprises has a threshold effect of knowledge stock.

The remainder of this manuscript is organized as follows: Section 2 measures the financing constraints of China logistics enterprises and analyzes the evolution trend of changes in their performance. Section 3 is data source and research design. Section 4 is the analysis of empirical results and discussion. Finally, Section 5 summarizes the main findings.

## Analysis of financing constraint measurement and corporate performance growth trend of logistics enterprises in China

### Data and variables

This paper selects listed companies in the transportation, warehousing and postal industries classified by the China Securities Regulatory Commission (CSRC) from 2010–2019 as research samples. In addition, companies that have been specially dealt with by the CSRC are excluded from the sample, mainly considering the abnormal financial problems of the companies and listed companies with more missing data. After the above sample processing, our dataset is from the Wind database of 101 listed logistics companies in China. Our data is processed using the STATA 16.0 software.

### Sample pre-grouping method and variable selection

According to the principle of binary logit regression, we first selects financial indicators to pre-divide all observation samples into two groups, among which the high financing constraint group is assigned a value of 1 and the non-high financing constraint group is assigned a value of 0. We sort the interest protection multiple from low to high, and select the top 33% of the sample companies as the high financing constraint group, with a financing constraint (LFC) value of 1, and the other non-high financing constraint group, with a financing constraint (LFC) assignment of 0 [32, 33].

We select five financial indicators as explanatory variables to construct a binary Logit model, namely Debt, ROE, NWC, DIV and RTR. Table 1 reports the specific meanings of each explanatory variables. Table 2 reports the data descriptive statistics.

**Table 1:** Variable definition.

Variables	Definition
**Debt**	Total liabilities/total assets
**ROE**	Net Profit/Shareholders’ Equity
**NWC**	The balance of the total current assets of the enterprise minus various current liabilities
**RTR**	The average number of times accounts receivable were converted to cash in a given period.
**DIV**	Cash dividends/total assets

**Table 2:** Summary statistics.

Variables	Min	Max	Mean	St. dev
**NWC**	-3.67e+10	1.68e+10	-8.28e+08	4.01e+09
**Debt**	1.0269	861.1787	46.3438	36.9340
**ROE**	-709.2647	64.4853	8.6687	25.3499
**RTR**	0	28996.46	80.0798	965.5247
**DIV**	-0.0018	2.8478	0.0661	0.2564

### Model and regression results

After selecting explanatory variables, we construct the following binary Logit regression model.


$$LFC_{it}=\beta_{0}+\beta_{1}*NWC_{it}+\beta_{2}*Debt_{it}+\beta_{3}*ROE_{it}+\beta_{4}*RTR_{it}+\beta_{5}*DIV_{it}+\varepsilon_{it}$$ (1)

The model is processed for heteroscedasticity, autocorrelation and regressed in parallel. Table 3 reports the regression results. From the sign of the regression coefficient, it can be seen that similar to the previous research conclusions, the financing constraint is negatively correlated with NWC, Debt, RTR, DIV, and positive correlation with ROE. The p-value of the ROE regression coefficient is 0.011, the p-value corresponding to the DIV regression coefficient is 0.001, and the p-value of other variables is 0.000, which is less than 0.05, indicating that the regression coefficients of all variables have passed the significance test. Based on the regression results of the above Logit model, we construct the following financing constraint index by using the regression coefficients of five variable indicators [34]. A large index value indicates a high degree of financing constraints. Table 4 reports the results of the LFC index calculations.


$$LFC_{it}=-3.4621-1.20e-10NWC_{it}+0.0760Debt_{it}-0.0536ROE_{it}-0.01026RTR_{it}-3.1161DIV_{it}$$ (2)

**Table 3:** Regression results.

LFC	Coef.	Robust Std. Err.	z	P>z	[95% Conf.	Interval]
**NWC**	-1.20e-10***	2.42e-11	-4.96	0.000	-1.68e-10	-7.27e-11
**Debt**	0.0756***	0.0065	11.63	0.000	0.0631806	0.0888
**ROE**	-0.0536**	0.0210	-2.55	0.011	-.0948089	-.0124469
**RTR**	-.01026***	0.0029	-3.58	0.000	-.0158638	-.0046469
**DIV**	-3.1161***	0.9023	-3.45	0.001	-4.884627	-1.347653
**cons**	-3.4621***	0.2946	-11.75	0.000	-4.039484	-2.884725
r^2^_a	0.3037					
**Log pseudolikelihood**	-434.30					

Note: * ≤ 0.10, ** ≤ 0.05, *** ≤ 0.01.

**Table 4:** The LFC index calculations.

Year	LFC Mean					
	Total samples	East	Middle	West	State-owned enterprises	Private enterprises
2010	2.338429	1.657035	2.80179	4.783749	2.450997	1.100183
2011	1.996177	1.807011	-1.44555	8.621644	2.122078	1.751945
2012	-1.01224	2.077665	-19.9521	2.856435	-1.45615	2.930892
2013	3.671349	2.874311	7.932797	2.597359	4.031001	3.124287
2014	2.457122	2.242225	2.852695	2.977605	2.664814	2.454132
2015	2.462136	2.239366	2.871514	3.360493	2.641621	2.77407
2016	2.538046	2.228012	3.707075	2.69208	2.751698	2.544629
2017	2.353509	2.33367	2.066882	2.615859	2.490615	3.030743
2018	2.484532	2.468315	2.562295	2.441475	2.589176	3.640507
2019	2.463797	2.503612	2.415121	2.459483	2.526516	4.061701

Note: The amount of data is large, so only the sample mean is listed here, you can contact the author if necessary.

### Dynamic evolution and forecasting of financing constraints and corporate performance growth of logistics enterprises in China

Using the results of the regression, we calculate the financing constraint index for all companies for each year. We use ROA indicators to measure the growth of corporate performance. The Kernel density and Markov chain model are used to analyze the dynamic evolution of financing constraints and the growth of corporate performance of logistics enterprises in China, and the trend is predicted.

**Fig 1:**
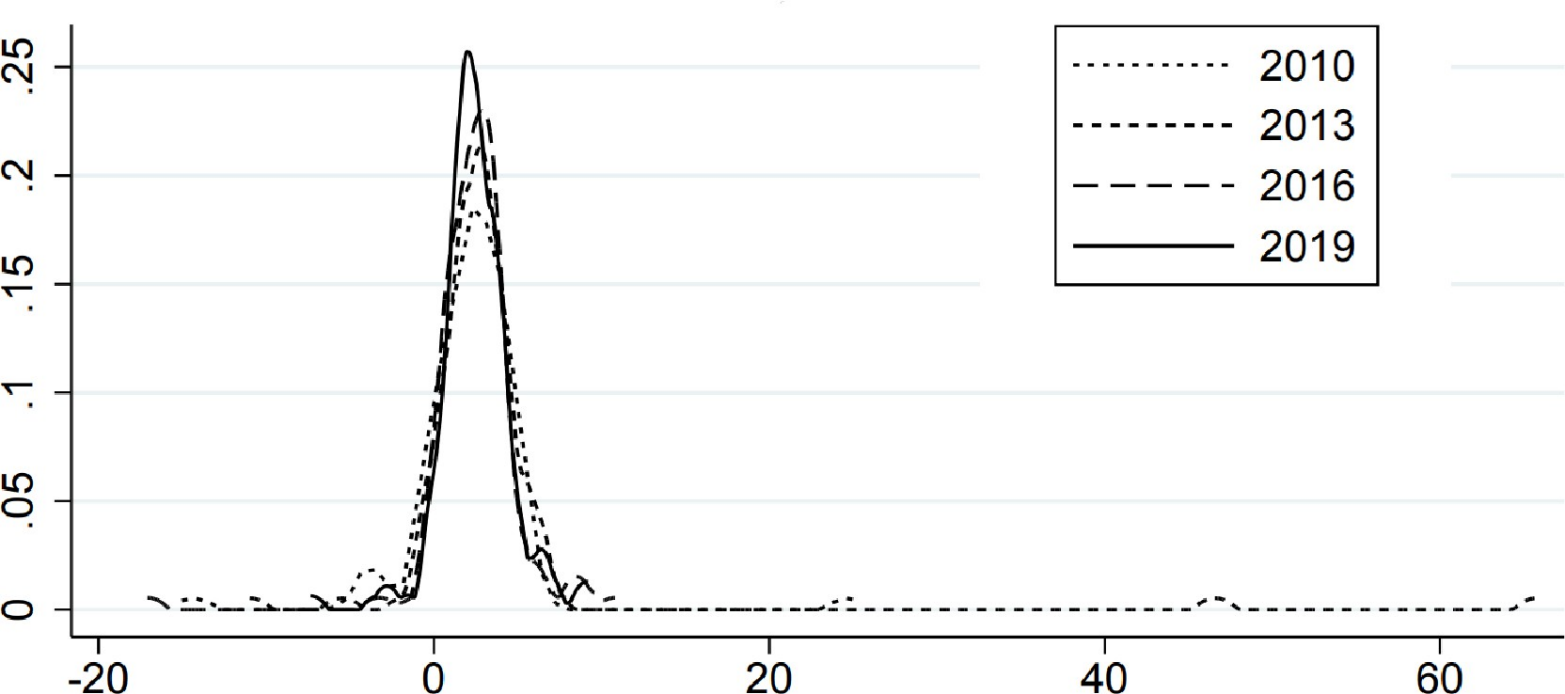
Kernel density estimation of corporate performance growth.

**Fig 2:**
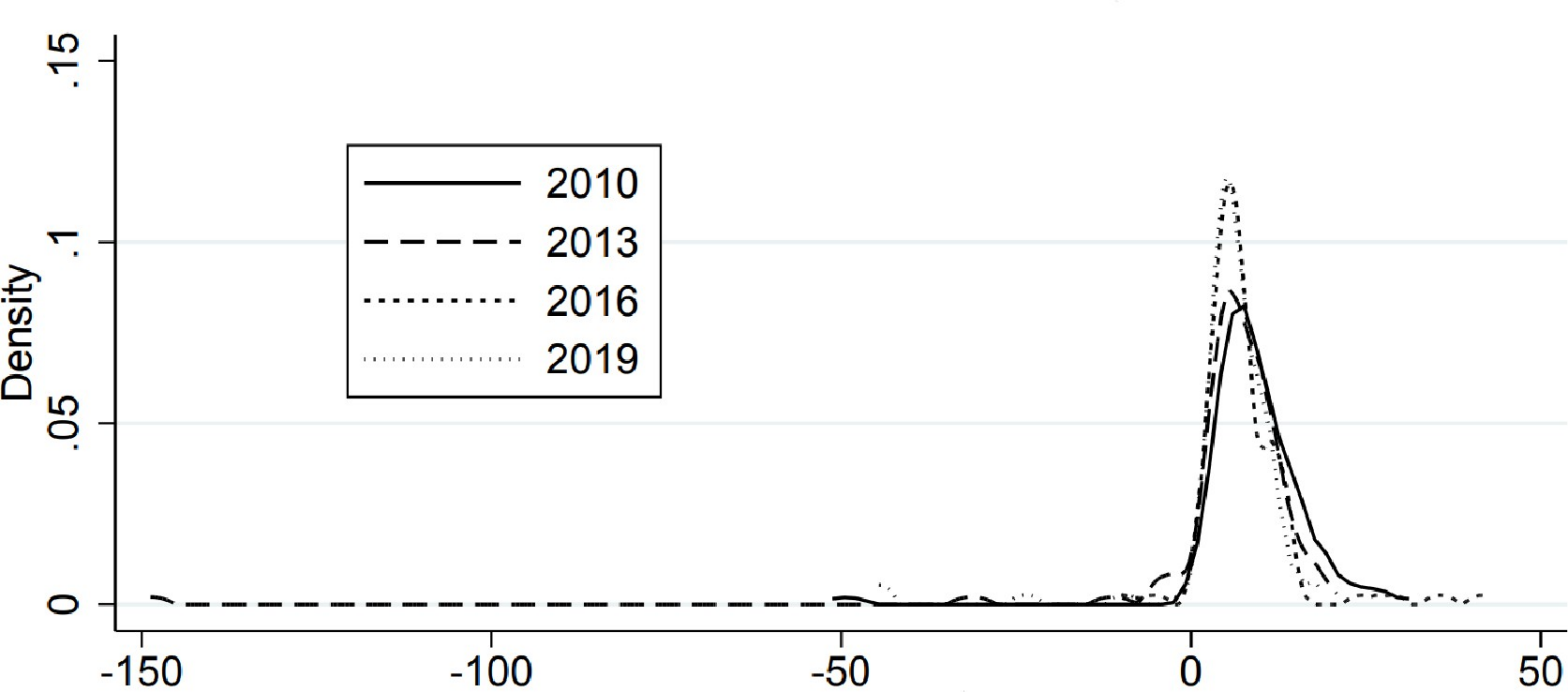
Kernel density estimation of corporate performance growth.

The kernel density estimation method is used to investigate the dynamic evolution of the degree of financing constraints and corporate performance changes of logistics enterprises in China during the sample period. The dynamic distribution characteristics of financing constraints and enterprise performance in different periods were compared. Figs 1 and 2 show the results of kernel density estimation (Figs 1 and 2).

The dynamic evolution characteristics of the degree of financing constraints of logistics enterprises in China mainly include the following aspects. First, in the sample period of 2010~2019, the kernel density estimation curve shifted to the left, indicating that the degree of financing constraints of logistics enterprises in China is decreasing from high to low. With the change of time, the financing constraints of China logistics enterprises have been alleviated to a certain extent. Second, the distribution pattern of the crest shows "flat-steep" fluctuation trend. Compared with 2010, 2013 and 2016, the peak of the kernel density curve of the financing constraint degree of logistics enterprises in China in 2019 is steeper. The center of the density function curve shifts to the left, and the peak value increases sequentially. This shows that the spatial gap in the degree of financing constraints of logistics enterprises in China is not obvious. Third, the number of peaks did not show a significant change, and the peaks of the kernel density curve became steeper and steeper. It shows that the degree of financing constraints of logistics enterprises in China has not shown obvious polarization.

The dynamic development characteristics of the performance of China logistics enterprises mainly include the following aspects. First, in the sample period 2010~2019, the left shift of the kernel density estimation curve is small and not obvious. It shows that with the passage of time, the performance of China logistics enterprises has not changed significantly and has not been significantly improved. Second, from the distribution pattern of the peak, the crest change shows a "flat-steep" fluctuation trend. Compared with 2010 and 2013, the peak of the core density curve of China logistics enterprise performance in 2016 and 2019 is steeper. The center of the density function curve shifts to the left, and the peak increases in turn. This demonstrates that there is no obvious spatial gap in the performance of China logistics enterprises. Third, from the perspective of the number of peaks, the number of peaks during the investigation period displays s an insignificant change trend, and the peaks of the kernel density curve are getting steeper and steeper. It shows that the performance of China logistics enterprises has not shown obvious polarization.

It can be seen that with the passage of time, the financing constraints of China logistics enterprises have been alleviated to a certain extent. And the corporate performance has not improved much and has remained almost unchanged. However, both show a "flat-steep" fluctuation trend and no obvious polarization.

In order to predict the financing constraint level and the long-term trend of corporate performance growth of logistics enterprises in China, this paper uses the traditional Markov chain method to carry out analysis. First of all, the financing constraint index and enterprise performance level of logistics enterprises in China are discretely divided into four levels according to the quartile method, which are low level (I), medium low level (II), medium high level (III) and high level (IV). Then, the Markov chain transfer probability matrix of China logistics enterprise financing constraint index and enterprise performance level is calculated with a lag of one year, and the results are shown in Tables 5 and 6.

**Table 5:** Financing constraint horizontal Markov model transfer probability matrix (k = 4).

t/t+1	Ⅰ	Ⅱ	Ⅲ	Ⅳ
**Ⅰ**	0.747475	0.191919	0.050505	0.010101
**Ⅱ**	0.166667	0.638095	0.180952	0.014286
**Ⅲ**	0.017544	0.192982	0.600877	0.188596
**Ⅳ**	0.007326	0.021978	0.14652	0.824176

**Table 6:** Corporate performance horizontal Markov model transfer probability matrix (k = 4).

t/t+1	Ⅰ	Ⅱ	Ⅲ	Ⅳ
**Ⅰ**	0.743697	0.193277	0.054622	0.008403
**Ⅱ**	0.267206	0.562753	0.17004	0
**Ⅲ**	0.023697	0.170616	0.805687	0
**Ⅳ**	0.5	0	0.5	0

For China logistics listed enterprises, 74.7475% of the financing constraint index of enterprises will remain at a low level after one year, and 19.1919% of enterprises will transfer the financing constraint index to the medium and low level after one year. The probability of the financing constraint index jumping forward to a medium-high level and a high level is 5.0505% and 1.0101%, respectively. One year later, 63.8095% of the financing constraint index of logistics enterprises remained unchanged at a medium and low level. The probability of the financing constraint index of logistics enterprises shifting upward to a medium and high level is 18.0952%. The probability of the financing constraint index of logistics enterprises moving to a high level in a positive leap is 1.4286%, and 16.6667% of enterprises moving to a low level. 60.0877% of enterprises will remain at a medium and high level after one year. 18.8596% of the financing constraint index of logistics enterprises will move up to a high level. The probability of logistics enterprise financing constraint index moving down to a medium and low level is 19.2982%, and the probability of jumping to a low level is 1.7544%. 82.4176% of enterprises remain at a high level of financing constraint index after one year, and the probability of logistics enterprise financing constraint index moving down to a medium and high level is 14.652%. The probability of negative jump transfer of the financing constraint index of logistics enterprises to the middle and low levels was 2.1978% and 0.7326%, respectively.

From the above analysis results, it can be seen that in the Markov transfer probability matrix of China logistics enterprise financing constraint index, the transfer probability on the diagonal is much greater than the probability on the non-diagonal. When the probability of maintaining the original level is greater than the probability of transfer, there is a club convergence at that level. Therefore, there is an obvious phenomenon of "club convergence" in the financing constraints of logistics enterprises in China, and the probability of low-level convergence and high-level convergence is large, 74.7475% and 82.4176%, respectively. The probability of convergence at the low and medium and high levels is small, 63.8095% and 60.0877%, respectively. The probability that the financing constraints of China logistics listed enterprises will remain stable is at least 60.0877%. It shows that there is a "Matthew effect" in the financing constraints of China logistics listed enterprises that the rich are richer and the poor are poorer.

74.3697% of China listed logistics companies still maintain a low level of corporate performance after one year. 19.3277% of enterprises will shift upward to a medium and low level after one year. The probability of positive leapfrogging shift in corporate performance to a medium and high level and a high level is 5.4622% and 0.8403%, respectively. The probability that the performance of logistics enterprises will remain unchanged at a medium and low level is 56.2753%. The probability of the performance of logistics enterprises shifting upward to a medium and high level is 17.004%. The probability of a positive jump in the performance of logistics companies to a high level is 0%. 26.7206% of enterprises will move to a low level. 80.5687% of logistics enterprises still maintain a medium and high level after one year. No logistics company performance will shift upwards to a high level. The probability of the performance of logistics enterprises shifting down to the medium and low level is 17.0616%. The probability of a jump to a low level is 2.3697%.0% of logistics companies still maintain a high level of business performance after one year. The probability of the performance of logistics enterprises shifting down to a medium and high level is 50%. The probability of negative jump shift in the performance of logistics enterprises to the middle and low levels and low levels is 0% and 50%, respectively. From the above analysis results, it can be seen that unlike the Markov transfer probability matrix of China logistics enterprise financing constraint index. The performance of China logistics enterprises has shifted from a high level to a low level. And the transfer from low levels to high levels is almost non-existent. It shows that it is difficult for China logistics enterprises to achieve and maintain a high level of corporate performance.

## Data source and research design

### Variables and data source

The performance growth of logistics enterprises (ROA) is selected as the Dependent variable, and the financing constraint (LFC) is used as the argument to construct an econometric model. Considering the impact of other factors other than financing constraints on the performance growth of logistics enterprises, we selected the shareholding ratio of the top ten shareholders (SRTTS), enterprise scale (SCA), working capital turnover ratio (WCTR) and enterprise age (AGE) as the control variables, and selected the knowledge stock (INT) as the threshold variable. Considering the economic value of knowledge stock and the availability of data, the intangible assets of enterprises are used to measure the level of knowledge stocks of enterprises [26]. Therefore, this paper uses the total intangible assets of listed companies at the end of the period to measure the knowledge stock of enterprises, and takes the logarithm of them. our dataset is from the Wind database of 101 listed logistics companies in China. Table 7 reports the data descriptive statistics.

**Table 7:** Summary statistics.

Variable	mean	sd	min	max
**ROA**	8.2114	25.5667	-199.5257	727.5291
**LFC**	2.1489	10.4851	-296.7356	65.7556
**INT**	18.62794	4.708351	0	24.64365
**AGE**	11.00	7.534	0	27
**WCTR**	13.4919	43.2202	0.0656	742.0756
**SCA**	22.9096	1.5041	18.4881	26.4489
**SRTTS**	64.2849	16.6125	7.3200	100.0100

In order to avoid the influence of multicollinearity of variables on the regression results, this paper conducted VIF test on the above variables. It can be seen from Table 8 that the VIF of each variable is less than 1.5, and there is no problem of multicollinearity.

**Table 8:** VIF test.

Variable	VIF	1/VIF
SRTTS	1.36	0.732873
SCA	1.32	0.757742
AGE	1.25	0.799979
LFC	1.01	0.990535
WCTR	1.01	0.993475
Mean VIF	1.19	

### Research design

Reference is made to the study of Yu and Xu [35]. We construct a panel data model to empirically test the research, and the specific model is as follows.


$$ROA_{it}=\beta_{0}+\beta_{1}LFC_{it}+\beta_{2}WCTR_{it}+\beta_{3}LEV_{it}+\beta_{4}SCA_{it}+\beta_{5}AGE_{it}+\beta_{6}SRTTS_{it}+\varepsilon_{it}$$ (3)

Referring to the relevant research of Hansen [36], we assume that the impact of financing constraints on the performance growth of logistics enterprises has a threshold effect of knowledge stock. The specific model is as follows.


$$ROA_{it}=\alpha_{0}+\alpha_{1}WCTR_{it}+\alpha_{2}LEV_{it}+\alpha_{3}SCA_{it}+\alpha_{4}AGE_{it}+\alpha_{5}SRTTS_{it}+\beta_{1}LFC_{it}I(INT<h_{1})+\beta_{2}LFC_{it}I(h_{1}<INT<h_{2})+\cdots +\beta_{n+1}LFC_{it}I(INT>h_{n})+\varepsilon_{it}$$ (4)

Where:

*ROA_it_* Stands for return on total assets, which is equal to the ratio of the company’s net profit plus interest expense to the average total assets, which is used to measure the performance growth of logistics enterprises.

*LFC_it_* Displays for financing constraints of enterprises.

*WCTR_it_* Indicates the working capital turnover ratio of the enterprise.

*LEV_it_* Is calculated by using the ratio of the total liabilities of the enterprise to the total assets. The proportion of corporate debt is large, and the cost of repaying debt interest is high.

*SCA_it_* Represents the scale of the enterprise, measured by the logarithm of the total assets of the enterprise.

*AGE_it_* Indicates the number of years from which a company went public to t year. Generally speaking, the longer a company goes public, the more standardized the corporate governance is.

*SRTTS_it_* Demonstrates the shareholding ratio of the top ten shareholders and measures the strength of the top ten shareholders controlling the company.

*LFC_it_* Indicates the financing constraints of the enterprise.

I(·) Is a schematic function, *INT* is the stock of knowledge, n is the number of thresholds, *h*_*1*_,*h*_*2*_…*h*_*n*_ is the threshold value.

*ε_it_* Is a random perturbation term.

## Empirical results and discussion

### Baseline results

We used panel data and selected a fixed-effect model for regression analysis after Huasman test. Table 9 displays the results of the Baseline results. The regression coefficient of financing constraint (LFC) is significantly negative, indicating that financing constraint inhibits the performance growth of logistics enterprises. This result shows that the logistics industry is facing a serious financing bottleneck, and also indicates that the multi-level capital market needs to be further improved. The high-quality development of the logistics industry needs to be supported by social funds. The expansion of enterprise scale can provide a better development environment for the logistics industry to achieve technological innovation and form economies of scale. In fact, logistics companies have economies of scale, which is verified in the regression results in Table 9. The SCA coefficient is significantly positive, indicating that the increase in the scale of the enterprise is conducive to the growth of the performance of logistics enterprises. The shareholding ratio of the top ten shareholders is significantly positive, indicating that the shareholding ratio of shareholders has a certain role in improving the performance of enterprises. The LEV coefficient is significantly negative, and the larger the corporate debt ratio, the higher the interest cost paid by the enterprise to repay the debt, and the lower the corporate performance.

**Table 9:** Empirical results.

Variables	(1)	(2)	(3)
	ROA	ROE	ROA
LFC	-0.025**	-0.002*	
	(0.012)	(0.001)	
KZ			-1.619***
			(0.423)
SHRCR	0.064	0.007	0.148***
	(0.049)	(0.006)	(0.048)
WCTR	0.001	0.000	0.001
	(0.003)	(0.000)	(0.002)
AGE	-2.318***	-0.291***	-1.281*
	(0.645)	(0.088)	(0.740)
SCA	2.240***	0.305**	0.774*
	(0.792)	(0.130)	(0.428)
Constant	-41.881**	-4.696*	-16.479**
	(17.277)	(2.748)	(8.214)
Observations	479	466	418
R-squared	0.108	0.088	0.362

Note:Robust standard errors in parentheses. *** p<0.01, ** p<0.05, * p<0.1

### Robustness checks

In this subsection, we show that our main findings are robust to alternative specification choices along the following dimensions.

### Replace the ROA variable

In order to ensure the accuracy of the empirical results, this paper replaces the explained variables. Replace ROA with ROE to check the robustness of the main model. Table 9 shows that the results are still significant after substitution.

### Replace the LFC variable

For the accuracy of the empirical results, this paper replaces the construction method of the financing constraint index, replaces the LFC index with the KZ index, and tests the robustness of the model.

Drawing on the research of Kaplan and Zingales [37], we use Chinese listed logistics companies as samples to construct the KZ index to measure the degree of financing constraints. Specifically, we construct the KZ index according to the following steps: (1) For each year of the full sample, calculate net operating cash flow/total assets of the previous period (CF_it_ /A_it−1_), cash dividends/total assets of the previous period (DIV_it_ /A_it−1_), cash holdings/total assets of the previous period (C_it_ /A _it−1_), asset-liability ratio () and Tobin’s Q (Q_it_). If CF_it_ /A_it−1_ is lower than the median, kz_1_ takes1, otherwise it takes 0. If DIV_it_ /A_it−1_ is lower than the median, kz_2_ takes 1, otherwise it takes 0. If C_it_ /A _it−1_ is lower than the median, kz_3_ takes 1, otherwise it takes 0. If LEV_it_ is higher than the median, kz_4_ takes 1, otherwise it takes 0.If Q_it_ is higher than the median, kz_5_ takes 1, otherwise it takes 0. (2) Calculate the KZ index, let KZ = kz_1_ + kz_2_ + kz_3_ + kz_4_+ kz_5_. (3) Using Ordered Logistic Regression, the KZ index is used as the dependent variable to perform regression on CF_it_/A_it-1_, DIV_it_/A_it-1_, C_it_/A_it-1_, LEV_it_ and Q_it_, and estimate the regression of each variable coefficient. (4) Using the estimated results of the above regression model, we can calculate the KZ index of the degree of financing constraints of each listed company. The larger the KZ index, the higher the degree of financing constraints faced by listed companies.

Table 9 shows that the results are still significant after replacing the core explanatory variables.

### Threshold regression results

First, this paper uses Stata 16.0 for panel threshold estimation. The 300-time Bootstrap method was used to test whether there was a threshold effect and the number of thresholds in the impact of financing constraints on the performance growth of logistics enterprises. The form of the panel sill model is determined by inspection. Table 10 reports the obtained F statistic values and their associated probability values. In this paper, the F-statistic analysis is carried out on the national sample, and the results show that the F-statistics of single threshold and double threshold reject the null hypothesis of “no threshold” and “only one threshold” at a significant level of 1%, respectively, and affirm that there is a double threshold for knowledge stock. The threshold test of knowledge stock was further carried out for different regions. The F-statistic in the eastern region rejects the “no threshold” at the 1% significant level, affirming the double threshold of knowledge stock. The central region also affirmed the double threshold. In the western region, there is no threshold. At the same time, the threshold value of knowledge stock is tested according to the nature of the enterprise. The results show that the F statistic of state-owned enterprises rejects the null hypothesis of “no threshold” and “only one threshold” at the significant level of 1%, and affirms that there are double thresholds for knowledge stock. Private companies have also affirmed the double threshold. Therefore, hypothesis 2 is validated. Based on the above analysis, the threshold value of the knowledge stock of the national sample, the eastern, central and western regions, state-owned enterprises and private enterprises was determined. Table 11 reports the specific thresholds.

**Table 10:** Threshold number test result.

Threshold variables	Sample	Threshold inspection	F	10% threshold	5% threshold	1% threshold
Knowledge stock	Total samples	Single threshold	48.12 ***	18.9448	24.8867	39.3762
		Double threshold	71.66 ***	16.1903	18.5302	23.2090
	East	Single threshold	43.63 ***	20.2630	26.1420	31.0356
		Double threshold	42.83 ***	18.2277	22.3901	31.5676
	Middle	Single threshold	67.06***	5.9309	7.5715	16.6337
		Double threshold	23.07 **	9.9306	11.1579	15.9063
	West	Single threshold	Does not exist			
		Double threshold				
	State-owned enterprises	Single threshold	35.210***	15.864	18.796	24.858
		Double threshold	41.090***	11.991	14.031	24.109
	Private enterprises	Single threshold	56.910***	15.987	18.616	34.004
		Double threshold	37.710***	12.173	16.714	19.679

Note: * ≤ 0.10, ** ≤ 0.05, *** ≤ 0.01.

**Table 11:** Threshold estimation results.

Threshold variables	Sample	Threshold value r1 Estimates	Threshold value r2 Estimates
Knowledge stock	Total samples	8.9425	22.2609
	East	12.2243	22.1707
	Middle	21.4213	13.4796
	West	Does not exist	
	State-owned enterprises	13.063	14.770
	Private enterprises	15.0698	20.4910

Based on the double threshold of the test model, after estimating the threshold values r1 and r2, this paper tests the panel model. From the national sample observation, Table 12 shows that before and after the threshold, the impact of financing constraints on the performance growth of logistics enterprises has shown a restraining effect. When the knowledge stock is lower than 8.9425, the impact effect of financing constraints on the performance growth of logistics enterprises is not significantly negative, and the coefficient is -0.340. When the knowledge stock is between [8.9425 22.2609], the impact effect of financing constraints on the performance growth of logistics enterprises is significantly negative, with a coefficient of -1.647. When the knowledge stock is greater than 22.2609, the impact effect of financing constraints on the performance growth of logistics enterprises is significantly negative, and the coefficient is -0.546. In general, the impact of financing constraints on the performance growth of logistics enterprises has a restraining effect of first increasing and then decreasing.

**Table 12:** Total sample threshold regression results.

ROA	Coef.	Std. Err.	t	P>t	[95%Conf.Interval]	
**SHRCR**	-0.048***	0.013	-3.700	0.000	-0.074	-0.022
**SCA**	0.729***	0.095	7.630	0.000	0.539	0.918
**AGE**	0.403	0.448	0.900	0.371	-0.485	1.291
**WCTR**	0.013**	0.005	2.670	0.009	0.003	0.022
**LFC (INT< 8.9425)**	-0.340*	0.173	-1.960	0.052	-0.683	0.004
**LFC(8.9425<INT<22.2609)**	-1.647***	0.226	-7.300	0.000	-2.094	-1.199
**LFC(INT>22.2609)**	-0.546**	0.210	-2.600	0.011	-0.963	-0.129
**_cons**	-3.691**	1.559	-2.370	0.020	-6.785	-0.597

Note: * ≤ 0.10, ** ≤ 0.05, *** ≤ 0.01.

Second, Tables 13 and 14 report panel threshold results for subregions. When the knowledge stock in the eastern region is lower than 12.2243, the impact effect of financing constraints in the eastern region on the performance growth of logistics enterprises is negative, and the coefficient is -0.215. When the knowledge stock is between [12.2243 22.1707], the impact effect of financing constraints on the performance growth of logistics enterprises is significantly negative, with a coefficient of -1.554. When the knowledge stock is greater than 22.1707, the impact effect of financing constraints on the performance growth of logistics enterprises in the eastern region is significantly negative, with a coefficient of -0.695. In general, the impact of financing constraints on the performance growth of logistics enterprises in the eastern region has a restraining effect of first increasing and then decreasing. The knowledge stock in the middle region is less than 13.4796, and the impact of financing constraints on the performance growth of logistics enterprises is significantly negative, with a coefficient of -0.007. When the knowledge stock is between [13.4796 21.4213], the impact of financing constraints on the performance growth of logistics enterprises is also significantly negative, with a coefficient of -1.134. When the knowledge stock is greater than 21.4213, the impact effect of financing constraints on the performance growth of logistics enterprises is negative, and the coefficient is -2.504. Overall, the inhibiting effect of financing constraints on the performance growth of logistics enterprises in the central region is increasing.

**Table 13:** Eastern region threshold regression results.

ROA	Coef.	Std. Err.	t	P>t	[95%Conf.Interval]	
**SHRCR**	-0.043**	0.015	-2.780	0.007	-0.074	-0.012
**SCA**	0.662***	0.097	6.810	0.000	0.469	0.855
**AGE**	0.531	0.508	1.050	0.299	-0.480	1.542
**WCTR**	0.007**	0.003	2.230	0.029	0.001	0.012
**LFC (INT< 12.2243)**	-0.215	0.171	-1.260	0.211	-0.555	0.124
**LFC(12.2243<INT<22.1707)**	-1.554***	0.276	-5.630	0.000	-2.104	-1.004
**LFC(INT>22.1707)**	-0.695**	0.318	-2.190	0.032	-1.327	-0.062
**_cons**	-2.936*	1.514	-1.940	0.056	-5.950	0.077

Note: * ≤ 0.10, ** ≤ 0.05, *** ≤ 0.01.

**Table 14:** Central region threshold regression results.

ROA	Coef.	Std. Err.	t	P>t	[95%Conf.Interval]	
**SHRCR**	-0.079*	0.039	-2.000	0.069	-0.164	0.007
**SCA**	-3.358**	1.396	-2.410	0.033	-6.401	-0.316
**AGE**	-1.040	1.030	-1.010	0.333	-3.285	1.205
**WCTR**	0.011	0.017	0.680	0.511	-0.025	0.048
**LFC(INT<13.4796)**	-0.007	0.012	-0.560	0.585	-0.033	0.020
**LFC(13.4796<INT<21.4213)**	-1.134***	0.266	-4.260	0.001	-1.714	-0.553
**LFC (INT>21.4213)**	-2.504***	0.105	-23.770	0.000	-2.733	-2.274
**_cons**	92.777**	27.493	3.370	0.006	32.875	152.679

Note: * ≤ 0.10, ** ≤ 0.05, *** ≤ 0.01.

Finally, Tables 15 and 16 report the results of the different enterprise nature tests. When the knowledge stock of state-owned enterprises is lower than 13.063, the effect of financing constraints on the performance growth of logistics state-owned enterprises is positive, and the coefficient is -0.009. When the knowledge stock is between [13.063 14.770], the impact effect of financing constraints on the performance growth of logistics enterprises is significantly negative, with a coefficient of -1.841. When the knowledge stock is greater than 14.770, the impact effect of financing constraints on the performance growth of logistics state-owned enterprises is significantly negative, and the coefficient is -0.642. In general, the impact of financing constraints on the performance growth of logistics state-owned enterprises has a suppressive effect that first strengthens and then decreases. When the knowledge stock is less than 15.0698, the impact effect of financing constraints on the performance growth of logistics private enterprises is negative, and the coefficient is -0.043. When the knowledge stock is between [15.0698 20.4910], the impact of financing constraints on the performance growth of logistics private enterprises is significantly negative, with a coefficient of -2.656. When the knowledge stock is greater than 20.4910, the impact effect of financing constraints on the performance growth of logistics private enterprises is also significantly negative, with a coefficient of -1.403. In general, the impact of financing constraints on the performance growth of logistics private enterprises shows a restraining effect of first increasing and then decreasing. It can be seen from the results that the inhibition effect of financing constraints on the growth of enterprise performance of state-owned enterprises is weaker than that of private enterprises. The gap between the threshold value of the knowledge stock of state-owned enterprises is small, while the threshold of private enterprises is large.

**Table 15:** State-owned enterprises state-owned enterprises threshold regression results.

ROA	Coef.	Std. Err.	t	P>t	[95%Conf.Interval]	
**SHRCR**	-0.004	0.016	-0.260	0.793	-0.036	0.028
**SCA**	-0.602	0.729	-0.820	0.412	-2.053	0.850
**AGE**	1.242**	0.618	2.010	0.048	0.011	2.473
**WCTR**	0.010*	0.006	1.820	0.073	-0.001	0.022
**LFC (INT< 13.063)**	-0.009	0.475	-0.020	0.985	-0.954	0.937
**LFC(13.063<INT<14.770)**	-1.841***	0.254	-7.260	0.000	-2.345	-1.336
**LFC(INT>14.770)**	-0.642***	0.177	-3.640	0.000	-0.994	-0.291
**_cons**	19.505	16.128	1.210	0.230	-12.604	51.614

Note: * ≤ 0.10, ** ≤ 0.05, *** ≤ 0.01.

**Table 16:** Private enterprises state-owned enterprises threshold regression results.

ROA	Coef.	Std. Err.	t	P>t	[95%Conf.Interval]	
**SHRCR**	-0.025	0.023	-1.100	0.283	-0.072	0.022
**SCA**	0.481**	0.155	3.110	0.005	0.160	0.803
**AGE**	1.762**	0.658	2.680	0.014	0.393	3.130
**WCTR**	0.026**	0.009	2.850	0.010	0.007	0.045
**LFC (INT< 15.0698)**	-0.043	0.121	-0.350	0.728	-0.294	0.209
**LFC(15.0698<INT<20.4910)**	-2.656***	0.592	-4.490	0.000	-3.886	-1.425
**LFC(INT>20.4910)**	-1.403**	0.416	-3.370	0.003	-2.267	-0.538
**_cons**	1.841	1.379	1.330	0.196	-1.027	4.709

Note: * ≤ 0.10, ** ≤ 0.05, *** ≤ 0.01.

The above empirical results show that the impact of financing constraints on the performance growth of logistics enterprises in China has a restraining effect. This inhibition effect has a double threshold of knowledge stock. With the increase of knowledge stock, there is a trend of first increasing and then decreasing. Enterprises of different nature in our country have similar characteristics. Although logistics enterprises in different regions also have a double threshold of knowledge stock. However, the restraining effect of financing constraints on the performance growth of logistics enterprises in the central region has shown a growing trend with the continuous increase of knowledge stock. The above results may occur for the following reasons. First, with the increase of enterprise knowledge stock, in the early stage, because more funds need to be invested in the knowledge stock, it occupies more enterprise funds. As a result, enterprises face stronger financing constraints, which in turn gradually increase the inhibition effect on the growth of enterprise performance. From the perspective of the long-term development of enterprises, the transformation of knowledge stock will alleviate the financing constraints of enterprises to a certain extent. The inhibition effect on the growth of enterprise performance is gradually decreasing, and even improves the performance of enterprises. Second, the distribution of regional resources is uneven, and there are differences in the degree of economic development. Therefore, with the increase of enterprise knowledge stock, the central and western regions are facing more serious financing constraints.

## Discussion

Taking Chinese sample as an example, this paper explores the level of financing constraints and its dynamic evolution trend in the development of China logistics industry. It also analyzes the nonlinear impact mechanism of financing constraints on the performance growth of logistics enterprises, and provides a new path for improving the high-quality development of China logistics industry. Compared with the existing research, the research on financing constraints mainly focuses on real estate and Internet companies in the industry, and there are fewer researches on the logistics industry. At present, the research on logistics enterprises is mainly concentrated at the industry level, mainly from the perspectives of logistics efficiency, business performance, and strategic issues. Most of the existing research conclusions support that financing constraints negatively affect the daily investment and business activities of enterprises, and most studies believe that knowledge stock can promote enterprise performance. However, a small number of scholars found that the stock of knowledge will make the enterprise have path dependence, but it will not be conducive to the improvement of the innovation performance of the enterprise.

This study extends the existing literature, including analyzing the dynamic evolution trend of financing constraints and corporate performance levels in China logistics industry, and selecting capital stock as a threshold variable to analyze the nonlinear relationship between financing constraints and corporate performance growth. This study provides a new policy-making path for China logistics industry to achieve high-quality development at this stage. For the research on the level of financing constraints in China logistics industry, the results show that the level of financing constraints in China logistics industry has not been significantly alleviated, and there is a "Matthew effect". There is no obvious change in corporate performance, and it is difficult to achieve and maintain a high level of corporate performance. The influence of China logistics industry financing constraints on enterprise performance growth has a double threshold effect of knowledge stock, and there is inhibitory effect of first increase and then decrease. Observing the nonlinear relationship between financing constraints of Chinese logistics enterprises and the growth of enterprise performance by region, it is found that there is also inhibitory effect of increases first and then decreases in the eastern region. However, financing constraints in the central region have increasing inhibitory effect on the performance growth of logistics enterprises. Due to the large differences in economic development levels between eastern China and the central and western regions, the eastern region has a strong attraction for investment in the logistics industry in terms of regional advantages and development foundations. And it is more attractive to talents, so more new technologies and new products can be developed, and the knowledge stock of enterprises can be improved. For the economically underdeveloped provinces in the central and western regions, the opening-up efforts are still insufficient. In increasing investment in the field of logistics, it is necessary to improve the location conditions and focus on improving the infrastructure of the logistics industry. By improving the location conditions to increase the investment attractiveness of the logistics industry. At the same time, it should increase and guide the logistics industry capital to invest in smart logistics to improve labor efficiency. When easing the financing constraints faced by enterprises, it also increases the knowledge stock of enterprises. Distinguish the nature of enterprises and observe the nonlinear relationship between financing constraints of China logistics industry and corporate performance growth. The study finds that the financing constraints of state-owned enterprises and private enterprises have a threshold effect of knowledge stock on the performance growth of logistics companies, and both show inhibitory effect of increases first and then decreases. However, private enterprises have more serious financing constraints, which are inseparable from the low attractiveness of private enterprises to talents, resulting in a low threshold for enterprise knowledge stock. To promote enterprise development, it is necessary to take attracting and retaining talents as a key path to improve labor efficiency.

## Conclusions and outlook

This paper uses the binary logit model to construct a financing constraint index to measure the degree of financing constraint of logistics enterprises. Kernel density and Markov chain model are used to analyze the dynamic change characteristics of financing constraints and business performance of logistics enterprises in China. Secondly, a panel threshold regression model is constructed to analyze whether the impact of financing constraints on the performance growth of logistics enterprises has a threshold effect of knowledge stock. Then, the heterogeneity analysis is carried out from the aspects of regional and enterprise ownership attributes, the difference of test results is compared, and the impact of financing constraints on the performance growth of logistics enterprises is explored. The main conclusions are as follows.

First, the kernel density and Markov chain model are used to analyze the financing constraints and dynamic characteristics of corporate performance of logistics enterprises in China. The results show that the degree of financing constraints of logistics enterprises in China is decreasing from high to low. With the passage of time, the financing constraints of China logistics enterprises have been alleviated to a certain extent, and the spatial gap is not too obvious. And there is a "Matthew effect" in which the rich are richer and the poor are poorer. However, the performance of China logistics enterprises has not been significantly improved, and there is no obvious spatial gap and polarization. And it is difficult for China logistics enterprises to achieve and maintain a high level of corporate performance.

Second, financing constraints have obvious negative impacts on the performance growth of logistics enterprises. The development of the logistics industry requires the injection of social capital, and to solve the financing bottleneck of the logistics industry, it is necessary to alleviate the impact of financing constraints on the performance growth of logistics enterprises. Under the current economic situation, improving and developing a multi-level capital market is of great significance for promoting the high-quality development of logistics enterprises.

Third, the impact of financing constraints on the performance growth of logistics enterprises in China has a double threshold inhibition effect on knowledge stock. With the increase of knowledge stock, there is a trend of first increasing and then decreasing. Similar characteristics exist in enterprises of different nature in China. Although logistics enterprises in different regions also have a double threshold of knowledge stock, the financing constraints in the central region have a restraining effect on the operating performance of logistics enterprises. With the increasing stock of knowledge, there is a growing trend.

The reasons for the above results may be as follows. First, with the increase of the stock of corporate knowledge, more funds need to be invested in the stock of knowledge in the early stage, so more corporate funds will be occupied, which will cause the company to face stronger financing constraints. From the perspective of the long-term development of enterprises, the transformation of knowledge stock formation results will alleviate the financing constraints of enterprises to a certain extent. The inhibitory effect of financing constraints on the business performance of enterprises is gradually decreasing, and it may even improve the business performance of enterprises. Second, the distribution of regional resources is uneven and there are differences in the degree of economic development. Therefore, as the stock of corporate knowledge increases, the central and western regions face more serious financing constraints. We should take a cautious look at the inhibitory effect of financing constraints on the operating performance of logistics companies in different regions, and distinguish the inhibitory effects of different threshold ranges, so as to adjust the stock of corporate knowledge, and then contribute to the alleviation of corporate financing constraints and improve corporate performance. Third, it is relatively easy for SOEs to obtain external financing. In terms of enterprise listing, bank credit, and government investment, state-owned enterprises have a good credit foundation. It is easier to get external support. State-owned enterprises have large financing and debt scales, so the financing constraints they face are not too serious. Since the use of working capital conforms to the law of diminishing marginal production, it is difficult to make full use of enterprise funds with relatively abundant funds. Due to their small scale, poor operating performance, insufficient commercial credit ratings, and few mortgageable assets, private enterprises are vulnerable to market discrimination, making it difficult to obtain external financing from the financial market. As a result, enterprises face more serious financing constraints, forming a vicious circle of enterprise development.

The policy implications of this paper are as follows. To improve the high-quality development of China logistics industry, we can support the development of enterprises by formulating policies to ease financing constraints. And formulate relevant policies to encourage logistics enterprises to carry out innovative research and development, and improve the knowledge stock of enterprises. In turn, it will form a driving force to promote the high-quality development of logistics enterprises. Second, the government should increase the formulation of preferential policies for enterprises in the central and western regions of China, and encourage enterprises to select locations in the central and western regions. Through these, we can promote employment in the central and western regions, and then promote the economic development of the central and western regions. In addition, private logistics enterprises are encouraged to innovate, and relevant subsidy policies are formulated to provide technical support to promote enterprise development and improve enterprise performance.

With the progress of society and the rapid development of the economy and the Internet, Chinese laws and regulations related to financing are constantly improving. The market environment faced by enterprises is also constantly changing, and it is necessary to keep up with the pace of the times and strengthen theoretical study and research. Combining the latest financing theory with the actual situation of the enterprise, formulate a financing plan that is more in line with the development of the enterprise. Therefore, it promotes the development of enterprises, and then promotes the high-quality development of the logistics industry. In the process of writing this paper, there are also some shortcomings, which need to be further studied in the future. There is still room for companies to explore ways to ease corporate financing constraints. Due to limited time, this paper does not continue to study this part, which needs to be further expanded in the follow-up research.

## Supporting information

S1 Data (RAR)
